# Objective and Subjective Measurement of Cough in Asthma: A Systematic Review of the Literature

**DOI:** 10.1007/s00408-022-00527-0

**Published:** 2022-04-13

**Authors:** Joshua Holmes, Liam G. Heaney, Lorcan P. A. McGarvey

**Affiliations:** 1 Wellcome-Wolfson Institute for Experimental Medicine, Belfast, UK; 2 Wellcome-Wolfson Institute for Experimental Medicine, School of Medicine, Dentistry and Biomedical Sciences, Queen’s University, Belfast, UK

**Keywords:** Cough, Asthma, Objective, Subjective

## Abstract

**Background:**

The extent to which objective and subjective tools has been used to measure the characteristics and burden of cough in patients with asthma has not been reported.

**Objective:**

To review the large and extensive body of literature in asthma with the specific hypothesis that the characteristics of cough and clinical impact in this disease has only occasionally been studied.

**Methods:**

For this systematic review, we searched EMBASE and MEDLINE databases using a combination of MeSH terms for “cough” and “asthma” for studies published up to and including end of August 2021. Studies included for analysis were confined to those undertaken in adult patients (≥ 18 years) with asthma of any severity where any tool or method to specifically measure cough was employed.

**Results:**

Of 12,090 citations identified after our initial search, 112 full-text articles met criteria for inclusion in our analysis. We found that a broad range of objective and subjective measures have been used albeit with a lack of consistency between studies. Clinically important levels of cough associated with impaired health status were identified in patients with asthma.

**Conclusion:**

Although cough is a common symptom in asthma, the clinical features and accompanying healthcare burden have been studied infrequently. In studies where cough was measured, the methods employed varied considerably. A more consistent use of cough-specific measurement tools is required to better determine the nature and burden of cough in asthma.

## Introduction

Asthma is characterised by variable expiratory airflow limitation and a range of respiratory symptoms including wheeze, chest tightness, shortness of breath and cough. In clinical practice, physicians enquire as to the severity and impact of these symptoms and the levels of treatment required to control them. This approach helps to determine disease severity for the individual patient and helps inform a treatment plan to optimise asthma control. A number of assessment tools including the Asthma Control Questionnaire (ACQ) [1] and the Asthma Quality of Life Questionnaire (AQLQ) [2] have been developed and validated for use not only in routine clinical practice but as key efficacy endpoints in clinical trials of asthma therapy. However, these tools are not without limitations including a failure to measure the impact and burden of all asthma symptoms [3].

Cough was one such symptom, not routinely captured independently of other asthma symptoms in the existing tools that measure asthma control nor typically considered as an outcome variable in therapeutic trials. This is despite evidence to suggest that cough exerts significant burden for some asthmatic patients [4]. Cough is also more prevalent in those with more poorly controlled disease [5] and can have a substantial effect on a patient’s quality of life [6].

There are a number of tools developed to specifically measure the clinical impact of cough. These include patient-reported outcome measures which gather information directly from individual patients as to cough severity and its impact on overall quality of life. In addition, there are techniques to objectively record cough frequency, i.e. ambulatory cough monitoring, and to measure an individual patient’s cough response to inhaled tussive agents. The extent to which these objective and subjective measures of cough have been studied in patients with asthma is not known. To explore this further, a systematic review of the literature was undertaken with the specific hypothesis that despite the large and extensive body of literature in asthma, the characteristics of cough and clinical impact in this disease have only occasionally been studied.

## Methods

The aim of this study is to provide a descriptive systematic review of the extent to which cough has been assessed within the asthma literature. Due to the heterogeneity of the inclusion criteria and the number of different outcome measures compared in this study, a meta-analysis was not possible. The protocol for this systematic review was submitted to Prospero (ID CRD42017058711) and details have been provided below.

### Search Strategy and Selection Criteria

For inclusion of studies in this systematic review, we searched entries in both MEDLINE and EMBASE databases for studies published up to and including August 2021. The search strategy (as detailed in Table 1) used a broad range of search terms to ensure that all studies that were potentially eligible for data extraction were captured. In brief, a number of MeSH terms were used for “asthma” (as per a search strategy for asthma studies developed by the Cochrane Airways Group [7]) in conjunction with the subject heading “cough” which allowed for a large and varied yield of studies, therefore ensuring that all relevant studies would be captured.

**Table 1:** Search strategy

Asthma search	Cough search
1. exp Asthma/	17. exp Cough/ (Search 1)
2. asthma$.mp	17. cough.mp (Search 2)
3. (antiasthma$ or anti-asthma$).mp	
4. Respiratory Sounds/	
5. wheez$.mp	
6. Bronchial Spasm/	
7. bronchospas$.mp	
8. (bronch$ adj3 spasm$).mp	
9. bronchoconstrict$.mp	
10. exp Bronchoconstriction/	
11. (bronch$ adj3 constrict$).mp	
12. Bronchial Hyperreactivity/	
13. Respiratory Hypersensitivity/	
14. ((bronchial$ or respiratory or airway$ or lung$) adj3 (hypersensitiv$ or hyperreactiv$ or allerg$ or insufficiency)).mp	
15. ((dust or mite$) adj3 (allerg$ or hypersensitiv$)).mp	
16. or/1-15	
***Search 1 (1949–2018):**** Asthma Search* **AND** *Cough (MeSH)*	
***Search 2 (2018–August 2021)****: Asthma Search* **AND** *Cough (keyword)*	

### Inclusion Criteria

1. Studies confined to adult asthmatic patients (age > 18 years).
2. Studies investigating asthma of any level of disease severity.
3. Studies that use any form of tool or method to specifically measure cough in an asthmatic population. Measurement tools of interest were those designed to measure cough as a standalone clinical outcome measure. This may include but is not limited to objective cough measurements (cough frequency monitoring or cough challenge testing) and subjective measures (cough-specific quality of life questionnaires and/or patient-reported outcome measurement tools).
4. All interventional, observational and qualitative studies were considered for data analysis providing all other criteria were met.

### Exclusion Criteria

1. Studies where cough is considered to exist primarily as a consequence of an existing co-morbidity (e.g. GORD, lung cancer, lung fibrosis).
2. Studies using a measurement tool not primarily designed to measure cough. For example, a questionnaire with sub-items relating to cough alongside a broader measure of respiratory symptoms or lung health status.
3. Prevalence studies, case reports or reviews.
4. Studies not reported in the language.
5. Studies not conducted in humans.

### Study Selection Procedure

**Fig. 1:**
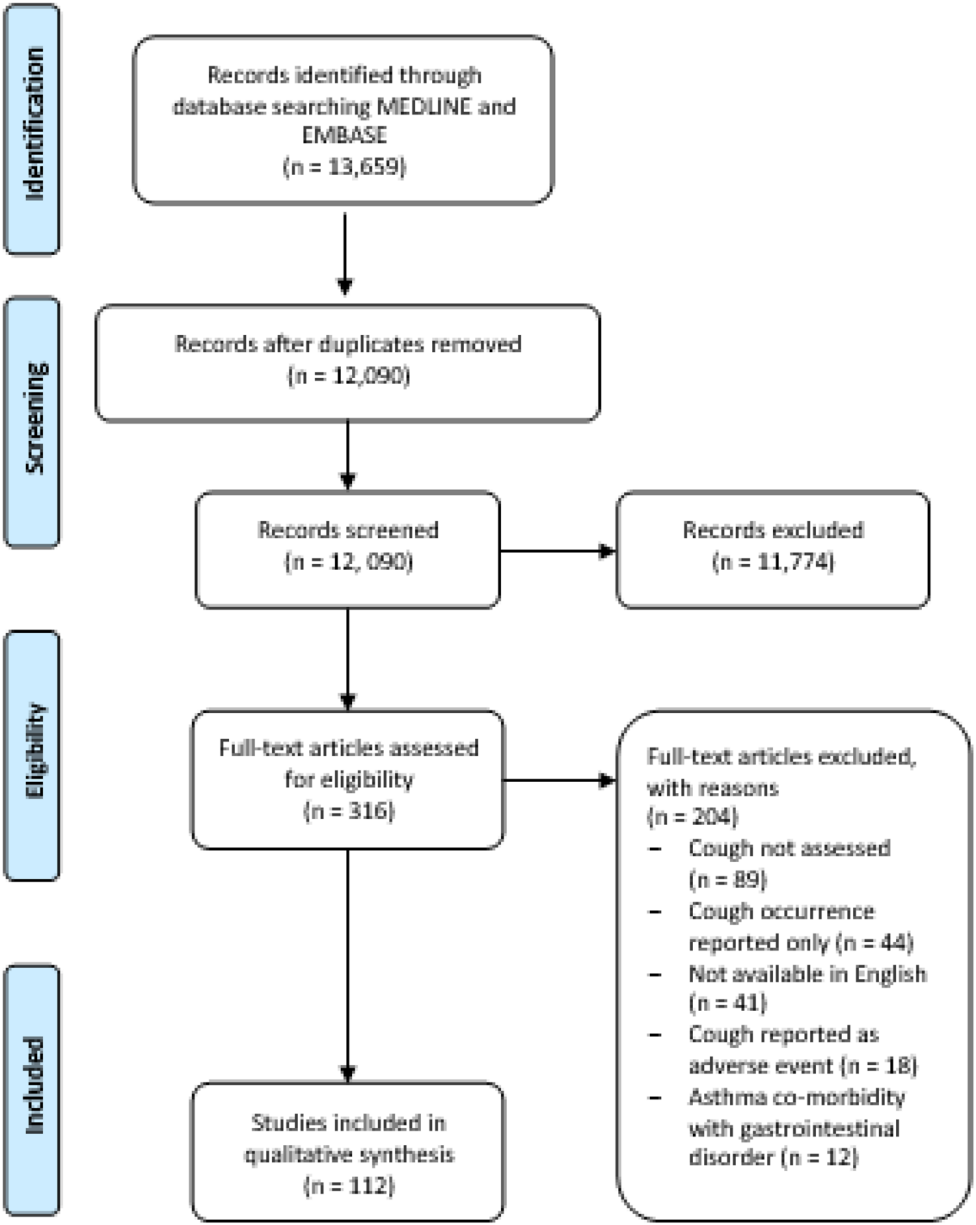
PRISMA flow diagram

An overview of the selection procedure, as per PRISMA guidelines, is detailed in Fig. 1. One reviewer (JH) initially identified and considered all titles and abstracts for potentially relevant papers. The following were removed; duplicate records, animal studies, reviews, case reports and non-English studies. In the final selection phase, two reviewers (JH and LMG) independently assessed the remaining papers. Any discordance on paper selection was resolved by discussion to achieve consensus.

### Data Analysis

A data extraction tool based on the work of the Cochrane Collaboration was used to collect data from each study. This allowed for assessment of study characteristics, methods, participant characteristics, cough measurement tools used and outcomes/results. Due to the heterogeneity of study characteristics, a meta-analysis was not performed.

Where appropriate, additional supplements were assessed during the data collection phase. Only those studies that use a specific cough measurement tool to assess cough in an asthmatic population were included for analysis.

### Risk of Bias Assessment

Methodological quality was assessed using a series of tools depending on the design of the study being reviewed. An adapted version of the Newcastle–Ottawa Scale (NOS) for nonrandomised trials (Online Supplement) was used to assess cross-sectional studies. The NOS allows for the assessment of the risk of bias across a number of domains (selection, comparability and outcome). The selection domain assesses sampling procedures (including sample size) and the quality of asthma diagnosis which helps to determine how representative the study participants were of the asthma population. The comparability domain assesses the extent to which confounding factors have been controlled for between study groups (i.e. are comparator groups well matched). Finally, the outcome domain assesses the quality and validity of measurement tools used within each study and helps inform as to whether the conclusions reached from each study are reliable and comparable. Studies were scored as very good (studies receiving nine or ten points), good (studies receiving seven or eight points), satisfactory (studies receiving five or six points) or unsatisfactory (studies receiving zero to four points).

As the remaining studies were randomised controlled trials (RCTs), the Joanna Briggs Critical Appraisal tool for Randomised Controlled Trials [8] was used (Online Supplement). Studies were awarded points for each “yes” answer to the questions within the tool and scored as very good (studies receiving 11 to 13 points), good (studies receiving 8 to 10 points), satisfactory (studies receiving 5 to 7 points) or unsatisfactory (studies receiving 0 to 4 points).

## Results

### Study Selection

Of the 12,090 citations retrieved from the initial search, 11,774 studies were removed following screening based on exclusion criteria. The remaining 316 full-text articles were assessed by 2 independent reviewers and agreement reached on all with 112 studies considered eligible for data analysis.

### Diagnosis of Asthma

There was variation in the diagnostic criteria for asthma reported in the selected studies which could be categorised as follows: reference to the use of national or international guideline-defined diagnostic criteria (*n* = 40); reliance on prior history of asthma and the presence of typical symptoms at time of study visit with confirmed airway hyperresponsiveness (*n* = 36); defined solely as ‘physician diagnosed’ (*n* = 22) and no record of any diagnostic criteria used (*n* = 14).

For cross-sectional studies, an adapted version of the NOS was used to assess quality of diagnosis (Appendix 1). Studies were awarded the highest score (2 points) if patients were diagnosed by guideline-defined criteria or following a detailed clinical assessment. Studies were also awarded 2 points if patients were established secondary care patients who were recruited following assessment of hospital records. 1 Point was awarded if patients were stated only as having “physician diagnosed” asthma or as having a known history of asthma. Finally, 0 points were awarded were patients self-reported an asthma diagnosis or were no diagnostic criteria were defined. In total, 52 (67.5%) of studies were awarded 2 points, 18 (23.4%) were awarded 1 point and 8 (10.4%) were awarded 0 points.

### Disease Severity

In 58 studies, asthmatics were recruited from single category of disease severity: mild asthma (*n* = 26), cough-variant asthma (CVA) (*n* = 25) and severe asthma (*n* = 7). A further 32 studies assessed patients across a range of disease severities: mild to severe asthma (*n* = 17), mild to moderate asthma (*n* = 11) and moderate to severe asthma (*n* = 4). The remaining studies (*n* = 22) reported no information on disease severity or there was no mention of specific diagnostic criteria other than a statement that asthmatic patients were recruited.

### Cough Measurement Tools

Many studies within this review reported findings from the use of more than one type of measurement tool. For the purpose of this review the range of tools employed have been categorised as follows: cough monitoring, cough reflex sensitivity testing, validated PRO measures and non-validated PRO measures.

When reviewing the use of cough measurement tools in RCTs compared to observational studies, there were some minor differences in the distribution of their use (Table 2).

**Table 2:** The proportion of each study type utilising different cough measurement tools either alone or in combination

	RCTs (*n* = 47)^a^ (%)	Observational studies (*n* = 65)^a^ (%)
Cough monitoring	9	20
Cough challenge testing	47	60
Validated subjective measures	19	29
Non-validated subjective measures	34	12

^a^Total percentages larger than 100% as a number of studies used more than one type of cough measurement tool

Additionally, the change in the use of cough measurement tools over time was assessed as follows: cough monitoring (1989–2020), cough challenge testing (1983–2021), validated subjective tools (1997–2020) and non-validated subjective tools (1982–2019). The only significant finding from this analysis is the introduction of studies using specialised ambulatory cough monitoring devices only from 2016.

### Cough Monitoring

There were 15 studies that used cough monitoring techniques to assess cough [9–23]. A full breakdown of the studies can be found within the Online Supplement.

The duration of cough recording time varied from 6 to 24 h. A range of cough frequency endpoints were reported which included total coughs, coughs per hour, cough seconds or cough events. Two additional studies monitored cough by qualitative analysis of cough sound signal [24] or by the assessment of flow dynamics and sound spectra of cough [25].

Marsden et al. reported in two studies [12, 13] that objective cough frequency was moderately correlated with subjective measures of asthma control and although cough frequency correlated closely with cough-related health status there was a poor association with subjective measures of cough severity. Higher cough frequencies were also observed in patients with asthma when compared to healthy controls [9, 16].

Two studies showed that there is an apparent diurnal variation in asthmatic cough frequency that appears to be independent of airway obstruction [9, 16]. Cough frequency was assessed in conjunction with cough challenge testing by Satia et al. [14, 15] who showed that an increased reflex sensitivity to capsaicin was associated with higher cough frequencies. These studies also showed evidence that increases in airway eosinophilia resulted in an increased sensitivity to capsaicin cough challenge and a subsequent increase in 24-h spontaneous coughing. A study from Wang et al. [17] also assessed cough frequency and cough reflex sensitivity and found that patients with severe asthma coughed considerably more during capsaicin cough challenge that those with mild/moderate asthma but did not report on the relationship between the two cough measures.

Three studies assessed the change in cough frequency following therapeutic intervention. Spector and Tan [11] showed that treatment with montelukast is effective in reducing cough frequency in patients with cough-variant asthma by 74%. Irwin et al. [10] also showed that beta-agonist therapy may not be as effective in reducing cough frequency in patients with cough-variant asthma but had an effect on reducing subjective cough severity. Finally, Faruqi et al. [23] showed that cough counts were significantly reduced in patients with severe asthma following 6 months treatment with mepolizumab.

Al-Khassaweneh and Abdelrahman [24] analysed cough sound signals and demonstrated that the “sound energy” of an asthmatic cough signal is greater than that of non-asthmatic cough, meaning that there is potential use of cough signals to potentially aid in the diagnosis of asthma. Finally, Piirila et al. [25] assessed the flow dynamics and sound spectra of cough in a number of respiratory conditions and found that peak expiratory flow is significantly lower in asthma than conditions, such as bronchitis. They also showed that, whilst the durations of the first spontaneous cough sound lasted longer than other conditions, patients with asthma subsequently had a lower number of additional spontaneous coughs.

### Measures of Cough Reflex Sensitivity

Cough reflex sensitivity testing was undertaken in 61 studies. Methodology varied widely with a broad range of tussive agents being used. This included capsaicin (*n* = 37), saline (*n* = 7), citric acid (*n* = 5), histamine (*n* = 4), tartaric acid (*n* = 3), mannitol (*n* = 2), sodium bicarbonate (*n* = 1) and sodium gluconate (*n* = 1) (a full list and breakdown of these studies is available in the Online Supplement). In most studies, a single chemical agent was studied, but in three studies two different agents were compared. Only one study assessed physical challenge based on mechanical stimulation of the trachea using stretch, compression and vibration techniques (*n* = 1).

Cough reflex sensitivity was reported to be heightened in patients with asthma compared to healthy controls in eight studies [14, 26–32]. However, in contrast to this, seven studies reported no difference in reflex sensitivity between these patient groups [33–39]. There was also evidence to suggest that cough reflex sensitivity may be heightened in patients with severe or uncontrolled asthma compared to those with mild and controlled asthma [17, 37, 40].

The relationship between cough reflex sensitivity and other cough measurement tools was investigated in ten studies. Heightened cough reflex sensitivity was associated with increases in cough severity [26, 41–45], a worsening cough-related quality of life [40, 43] and an increase in cough frequency [12, 14, 15, 43]. There was also evidence to suggest that cough reflex sensitivity is associated with measures of asthma control as measured by the Asthma Control Questionnaire (ACQ) [40] and the Asthma Control Test (ACT) [44].

There was evidence to suggest that changes in cough reflex sensitivity are not related to sputum eosinophilia [46] and levels of airway inflammation [47]. However, more recent evidence suggests that increases in airway eosinophilia may result in an increased cough reflex sensitivity to capsaicin which is also associated with increases in the amount of spontaneous coughing over 24 h [15].

Some studies provided evidence that treatment with leukotriene receptor antagonists (LTRAs) is effective at reducing cough reflex sensitivity in patients with cough-variant asthma [41, 48] but may not have the same impact in mild to moderate bronchial asthma [49]. A number of other studies also provided evidence that treatment with azelastine [50, 51], non-steroidal anti-inflammatories [52, 53], carbocysteine [54] and inhaled corticosteroid therapies [31, 36] are effective in reducing cough reflex sensitivity in mild to moderate asthma.

### Capsaicin Cough Challenge

Although capsaicin was the most commonly implemented tussive agent within the selected studies, the cough challenge methodology varied. Differences include the number of dilutions used, the minimum and maximum concentrations used, inhalation time (single breath vs tidal breathing) and the use of placebo doses. More consistency was observed for the endpoints of the cough challenge test with most studies using the concentration required to elicit 2 (*C*_2_) and/or 5 (*C*_5_) coughs as the point of termination of the test.

### Validated Patient-Reported Outcome Measures

19 Studies utilised at least one quality of life questionnaire to assess the impact of cough. The Leicester Cough Questionnaire (LCQ) (*n* = 18) was most commonly used with the Cough-Specific Quality of Life Questionnaire (CQLQ) (*n* = 2) and the Chronic Cough Impact Questionnaire (*n* = 1) was used infrequently. Additionally, a number of studies employed a Visual Analogue Scale to assess cough (*n* = 13). An overview of the studies is shown in the Online Supplement.

Cough-related quality of life (QOL) was associated with asthma symptom scores [55] and measures of asthma control and asthma-related QOL [40, 56]. Additionally, there was evidence to suggest that patients with uncontrolled asthma have a worse cough-related QOL compared to patients with controlled asthma [40]. Cough QOL was also associated with markers of airway inflammation [29, 40] but showed no significant association with sputum eosinophilia [56] or blood eosinophilia [57].

There were no significant differences observed in cough-QOL between patients with idiopathic chronic cough and patients with asthma and a chronic cough [57] or those with stable asthma [6]. Additionally, patients with asthma and cough reported higher frequencies of other asthma symptoms (wheeze, dyspnoea and chest tightness) compared to those patients without chronic cough [57].

Treating asthma patients with ICS [43], tiotropium [44], procaterol [58] and azithromycin [59] all resulted in improvements in cough-related QOL. Cough severity was shown to improve following treatment with montelukast [41, 60] and beta-agonist therapy [10]. Additionally, there was evidence to suggest that providing patients with additional interactive online advice and guidelines relating to their condition can help to improve cough-related QOL [61].

### Non-validated Patient-Reported Outcome Measures for Cough

Forty-two studies used a measurement tool which has not been validated to assess cough. A Likert scale or cough diary in conjunction with a validated cough measure was used in eighteen studies [9, 11, 26, 30, 31, 36, 42, 43, 45, 48, 50, 55, 58, 59, 62–65].

The remaining 24 studies used a measurement tool that not validated for the sole assessment of cough and consisted of Likert scales/cough diaries (*n* = 19), the European Community Respiratory Health Survey (ECRHS) (*n* = 3) and an interview (*n* = 1).

Studies that utilised a Likert scale or symptom diary were primarily used to monitor how the severity or frequency of cough changed in response to a treatment therapy. A number of studies reported reduction in cough frequency and severity scores with bronchodilator therapy [66–69]. Inhaled corticosteroid therapy was also reported to be effective either alone [70] or in combination with bronchodilators [71–73]. One study showed that increasing the dose of budesonide and formoterol therapy in the presence of cough alone significantly reduced the time to recover from symptoms compared to increasing the dose in the presence of dyspnoea or wheeze [74]. Additionally, treatment with clarithromycin [75], disodium cromoglycate [76] and LTRAs [68, 77] also showed efficacy in reducing cough symptom scores. Finally, through a questionnaire and follow-up interviews, one study [5] showed that the frequency of coughing is increased in patients with uncontrolled asthma compared to those with controlled asthma.

## Discussion

The aim of this review was to determine the extent to which the characteristics and clinical consequences of cough have been specifically addressed in studies undertaken in asthmatic subjects. We hypothesised that, despite the evidence for cough as an important symptom in asthma, it has been studied infrequently. Here, we report that, of the very large number of asthma studies within the existing literature, only a relatively small number have specifically addressed cough. The studies identified employed a range of objective and subjective instruments to measure cough although we noted little consistency in the choice of tool or standardisation in its use. Our review of the eligible studies indicates that clinically important levels of cough burden exists in subgroups of asthmatic patients, which is associated with impaired health status. We also report that cough is associated with impaired asthma control that is distinct from that recorded using current asthma control questionnaires. Below we discuss our interpretation of the analysis.

Subgroups of patients with asthma show considerable levels of cough burden and morbidity. Patients with asthma cough significantly more and experience a greater impairment in health status than healthy individuals [9, 16]. Some patients with asthma also have at least as much cough-related impairment and morbidity as those with idiopathic chronic cough [6, 57].

In the current literature, most studies of cough have been confined to patients with mild disease or CVA, with only a few conducted in patients with severe asthma. In addition, direct comparisons between asthma patients of differing disease severities have been studied infrequently. However, there was evidence that patients with uncontrolled asthma have a significantly greater cough frequency [13], worse cough-related QOL [40] and heightened cough reflex sensitivity [17, 37, 40] compared to patients with milder asthma. Furthermore, cough burden was not found to be associated with increased T2 inflammation suggesting that, from a clinical management perspective, it is not clear whether an escalation in dose of inhaled or oral corticosteroids may be an effective means of symptom control for all patients. It will be important to determine whether improvement in asthma control associated with the recently approved biological therapies is accompanied by a reduction in cough burden.

Our review of the literature suggests that cough measurement tools identify factors responsible for health burden and disease control that are quite distinct to that measured using instruments, such as the Asthma Control Questionnaire (ACQ) and the Asthma Quality of Life Questionnaire (AQLQ). These questionnaires do not specifically capture the impact of cough on disease control and their almost universal use in asthma studies over the last 30 years goes some way to explain the limited attention afforded to cough as a clinical problem.

A small proportion of studies within this review used cough monitoring to assess cough and in most cases ambulatory measurement of cough frequency was undertaken. Whilst it is apparent that an increased cough frequency is associated with poor asthma control and impaired health status there is a need to overcome technological issues including battery life and portability before it can be widely adopted in asthma clinical research.

Cough challenge testing was the most commonly used measurement tool in the studies we reviewed. However, we noted considerable heterogeneity in methodology including the choice of inhaled tussive agent or the delivery device used with little evidence of standardisation of the cough challenge protocol which hampered attempts to make comparisons between studies. It is apparent that cough reflex testing does not reliably distinguish between patients with asthma and healthy subjects calling into question its value in clinical research in asthma.

A number of the studies we analysed were therapeutic trials and used non-validated measures such as Likert scales to determine cough treatment efficacy [66, 67, 70, 75, 76, 78–80]. The use of currently available validated cough-specific patient-reported outcome measures in the design of future asthma trials should be encouraged.

As with all systematic reviews, there was variability in the quality of the studies reviewed. A number failed to provide a sample size calculation or provide information relating to predefined recruitment targets. Although there was variability in how asthma was defined or diagnosed, in the majority of selected studies reported, patients were recruited according to pre-specified national guidelines or following a systematic assessment of symptoms and physical measurements. We are reasonably confident that the findings of this review are representative of a general asthma population.

To conclude, this review has identified that, in the context of the vast asthma literature, cough has been underappreciated as a clinical problem. This is especially true in the setting of severe asthma, where disease burden is high, compounded by the effects of high dose oral steroids. There are a number of validated objective and subjective measures of cough which need to be incorporated into the design of asthma studies and clinical trials.
